# Characteristics of plastid genomes in the genus *Ceratostigma* inhabiting arid habitats in China and their phylogenomic implications

**DOI:** 10.1186/s12870-023-04323-7

**Published:** 2023-06-07

**Authors:** Yu-Juan Zhao, Jian Liu, Gen-Shen Yin, Xun Gong

**Affiliations:** 1 CAS Key Laboratory for Plant Diversity and Biogeography of East Asia, Kunming Institute of Botany, Chinese Academy of Sciences, Kunming, Yunnan, 650201 China; 2 Key Laboratory of Economic Plants and Biotechnology, Kunming Institute of Botany, Chinese Academy of Sciences, Kunming, 650201 China; 3 Yunnan Key Laboratory for Wild Plant Resources, Kunming, Yunnan, 650201 China; 4 Institute of Agriculture and Life Sciences, Kunming University, Kunming, 650214 China

**Keywords:** *Ceratostigma*, Plastid genome, Comparative analysis, Interspecific relationship, Plumbaginaceae

## Abstract

**Background:**

*Ceratostigma*, a genus in the Plumbaginaceae, is an ecologically dominant group of shrubs, subshrub and herb mainly distributed in Qinghai-Tibet Plateau and North China. *Ceratostigma* has been the focal group in several studies, owing to their importance in economic and ecological value and unique breeding styles. Despite this, the genome information is limited and interspecific relationships within the genus *Cerotastigma* remains unexplored. Here we sequenced, assembled and characterized the 14 plastomes of five species, and conducted phylogenetic analyses of *Cerotastigma* using plastomes and nuclear ribosomal DNA (nrDNA) data.

**Results:**

Fourteen *Cerotastigma* plastomes possess typical quadripartite structures with lengths from 164,076 to 168,355 bp that consist of a large single copy, a small single copy and a pair of inverted repeats, and contain 127–128 genes, including 82–83 protein coding genes, 37 transfer RNAs and eight ribosomal RNAs. All plastomes are highly conservative and similar in gene order, simple sequence repeats (SSRs), long repeat repeats and codon usage patterns, but some structural variations in the border of single copy and inverted repeats. Mutation hotspots in coding (Pi values > 0.01: *matK*, *ycf3*, *rps11*, *rps3*, *rpl22* and *ndhF*) and non-coding regions (Pi values > 0.02: *trnH*-*psbA*, *rps16*-*trnQ*, *ndhF*-*rpl32* and *rpl32*-*trnL*) were identified among plastid genomes that could be served as potential molecular markers for species delimitation and genetic variation studies in *Cerotastigma*. Gene selective pressure analysis showed that most protein-coding genes have been under purifying selection except two genes. Phylogenetic analyses based on whole plastomes and nrDNA strongly support that the five species formed a monophyletic clade. Moreover, interspecific delimitation was well resolved except *C*. *minus*, individuals of which clustered into two main clades corresponding to their geographic distributions. The topology inferred from the nrDNA dataset was not congruent with the tree derived from the analyses of the plastid dataset.

**Conclusion:**

These findings represent the first important step in elucidating plastome evolution in this widespread distribution genus *Cerotastigma* in the Qinghai-Tibet Plateau. The detailed information could provide a valuable resource for understanding the molecular dynamics and phylogenetic relationship in the family Plumbaginaceae. Lineage genetic divergence within *C*. *minus* was perhaps promoted by geographic barriers in the Himalaya and Hengduan Mountains region, but introgression or hybridization could not be completely excluded.

**Supplementary Information:**

The online version contains supplementary material available at 10.1186/s12870-023-04323-7.

## Background

*Cerotastigma* Bunge is a small genus falling within the tribe Plumbagineae (Plumbaginaceae), with eight species disjunctively distributed in tropical East Africa and East Asia [1]. South Tibet is the present distribution center for the genus [2]. There are five morphologically distinct species distributed in China, consisting of perennial shrubs, subshrubs and herbs, most of which are typically dominant species in arid environment in the Qinghai-Tibet Plateau *sensu lato* (QTP*sl*) [3]. For example, the geographic distribution of shrub *C. minus* shows a pattern that extends along xeric valleys of Yarlung Zangbo River and Hengduan Mountains across major gradients of elevation, *C. willmottianum* is an emblematic subshrub endemic to dry valleys mainly in Yunnan and Sichuan, and the cushion shrub *C. ulicinum* grows in open shrubland with a restrictive distribution range on the west QTP*sl* [2, 4]. As constructive and zonal species, they play crucial roles in sustaining the fragile arid ecosystems [2]. Additionally, all species are of great value in landscapes for their features of drought resistance, salinity tolerance as well as ease of cultivation [5]. And owing to their blue and violet flowers and long flowering time in autumn or winter, some are introduced as garden ornaments outside China [6]. *Cerotastigma* also contain a variety of chemical active ingredients such as plumbagin, which exhibits antitumor, anticancer and antibacterial activities [7, 8]. Therefore, *Cerotastigma* has important ecological and economic value.

To date, the genetic background and resources for *Cerotastigma* remain scarce. Several authors have studied the genus from the following aspects. Li proposed that the genus perhaps had an origin and evolved near Tethys during the early Tertiary [2]. From the breeding system perspective, the results of pollination experiments illustrated that *C*. *willmottianum* having distyly does not exhibit precise reciprocal herkogamy and was partially self-compatible but primarily outcrossing [9]. In our field survey, we found that the five species of *Ceratostigma* all have heterostyly as many species of Plumbaginaceae and the ratios of long-styled morphs and short-styled morphs exist in different populations with slight differences, which may reflect similar pollination strategies among the different species. Concerning the origins of distyly in Plumbaginaceae, Barrett et al. [10] constructed several models. Their results support the ideas of the more recent selfing avoidance model by D. & B. Charlesworth [11], in which distyly evolves from self-incompatible ancestors other than reciprocal herkogamy. Moreover, the medicinal and horticultural properties place the genus as target studies regarding establishing rapid propagation systems for obtaining plumbagin and developing polyploids used in landscapes [6]. Lastly, as we mentioned above, Plumbaginaceae including *Ceratostigma* species could survive in harsh environmental conditions like high salt media or heavy-metal-rich soils, and some researchers found a strong correspondence between these capacities and the secretory structures such as salt glands secreting a range of ions, which might have arisen as a means to avoid the toxicity or regulate ion concentrations within leaves [12]. However, up till now, the genome information of *Cerotastigma* is still limited, and no attempt has yet made to explore the interspecific relationships within this genus. Although several studies have incorporated molecular data to study the phylogeny of Plumbaginaceae, in which monophyly of *Cerotastigma* was confirmed by phylogeny of some representative genera or families [13–15], these inferences were derived from limited taxon of *Cerotastigma* or molecular sampling only comprising several plastid fragments, and thus limiting a comprehensive understanding of the interspecific relationships and divergence of *Cerotastigma*.

The plastid is a core organelle in plants and the utilization of complete plastome sequences has been widely accepted to resolve phylogenetic relationships at different taxonomic levels, such as all flowering plant families [16, 17], gymnosperms [18], tribe Cinnamomeae [19] and genus *Rhododendron* [20]. Recently, this strategy has worked for a range of taxa such as those having closely relationships with Plumbaginaceae (e.g., *Calligonum* and *Rheum* within Polygonoideae) [21, 22] and several representatives of *Limonium* belonging to Plumbaginaceae [23]. Despite this, for the species-rich and highly diverse family (27–29 genera) [24, 25], the plastid genomes information of representatives are less well-documented and need to be substantially complemented. To date, only one plastid genome of *Cerotastigma* plus six species from two other Plumbaginaceae genera *Limonium* [15, 23, 26–28] and *Plumbago* [15, 29] have been reported, which account for less than ca. 1% of the currently described ca. 650 Plumbaginaceae species [13, 24, 25]. Thus, to better understand the plastome structure and phylogenomics, it is essential to conduct the comparative analyses for *Cerotastigma* plastid genomes and assess the phylogenic relationships based on the plastid genomes sequences. In this study, we generated 14 plastomes data of *Cerotastigma* species. To obtain a comprehensive understanding of infrageneric phylogeny, the nuclear ribosomal DNA data was also used to construct phylogenetic tree. Our specific goals were as follows: (1) to compare the plastid structures within *Cerotastigma*; (2) to identify the variable hotspots as potential DNA markers for genetic variation studies; (3) to infer the phylogenetic relationships among *Cerotastigma* species within Plumbaginaceae. These results could provide new sources of information for phylogenic analyses of Plumbaginaceae and assist with the further population genomics of *Cerotastigma*.

## Results

### Chroloplast genome feature

The newly sequenced *Cerotasigma* plastid genomes were circular with the typical quadripartite structure with a large single copy (LSC), a small single copy (SSC) and two copies of inverted repeats (IR) regions (Fig. S1). The genome sizes were ranged from 164,076 bp in *C*. *minus* to 168,355 bp in *C*. *ulicinum*, and the IR regions were more variable (30,516−32,788 bp) than LSC (88,756−89,988 bp) and SSC regions (13,457−13,534 bp). The overall GC content was 37.3−37.7%, while the IR regions (40.7−41.4%) exhibited higher GC contents than those of both LSC (35.6−36.0%) and SSC regions (31.9−32.2%). Moreover, the five *Cerotastigma* plastoms were conserved in gene numbers and gene orders, with 82–83 protein-coding genes, 37 tRNA genes and eight rRNA genes (Table 1, Table S1). *C*. *ulicinum* had one more *rpl2* gene than four other *Cerotastigma* species in IRA (Fig. S1).

**Table 1:** Plastid genome characteristics of the five *Cerotastigma* species

Species	Locality ID	Total length (bp)	LSC (bp)	SSC (bp)	IRs (bp)	Total GC content (%)	LSC GC content (%)	SSC GC content (%)	IRs GC content (%)	No. of protein coding genes	No. of tRNA	No. of rRNA	GenBank Accession numbers
*C*. *griffithii*	ZN	164,622	89,828	13,530	30,632	37.6	35.9	32.0	41.2	82	37	8	OP954207*
*C*. *griffithii*	JC2	164,348	88,877	13,457	31,007	37.6	35.9	32.1	41.2	82	37	8	OP954206
*C*. *minus*	DQ	164,076	89,837	13,497	30,371	37.6	35.9	32.2	41.4	82	37	8	OP967035
*C*. *minus*	MK	164,987	89,988	13,477	30,761	37.7	36.0	32.2	41.3	82	37	8	OP967036
*C*. *minus*	NML	164,786	89,806	13,454	30,763	37.5	35.9	32.1	41.0	82	37	8	OP954210
*C*. *minus*	JC1	164,369	89,826	13,511	30,516	37.5	35.9	32.0	41.2	82	37	8	OP967033
*C*. *minus*	LOZ	164,488	89,558	13,520	30,700	37.5	35.8	32.1	41.1	82	37	8	OP954209
*C*. *minus*	BR	164,789	89,569	13,518	30,851	37.5	35.9	31.9	41.1	82	37	8	OP954208*
*C*. *minus*	MZ	164,182	89,418	13,464	30,650	37.6	35.9	32.1	41.2	82	37	8	OP967032
*C*. *plumbaginoides*	XW1	164,744	89,650	13,534	30,780	37.5	35.8	31.9	41.2	82	37	8	OP954204*
*C*. *plumbaginoides*	XW2	164,744	89,650	13,534	30,780	37.5	35.8	31.9	41.2	82	37	8	OP954205
*C*. *ulicinum*	AR	168,355	89,263	13,516	32,788	37.3	35.6	31.9	40.7	83	37	8	OP921765
*C*. *ulicinum*	JZ	167,576	88,756	13,492	32,664	37.3	35.6	32.0	40.8	83	37	8	OP921766*
*C*. *willmottianum*	KR	164,164	89,532	13,482	30,575	37.5	35.9	32.1	41.2	82	37	8	OP967034*

Note: The five plastid genome used for compared analyses were indicated by asterisks with GenBank accession numbers

### Comparisons of border and divergent hotspot identification analysis

**Fig. 1:**
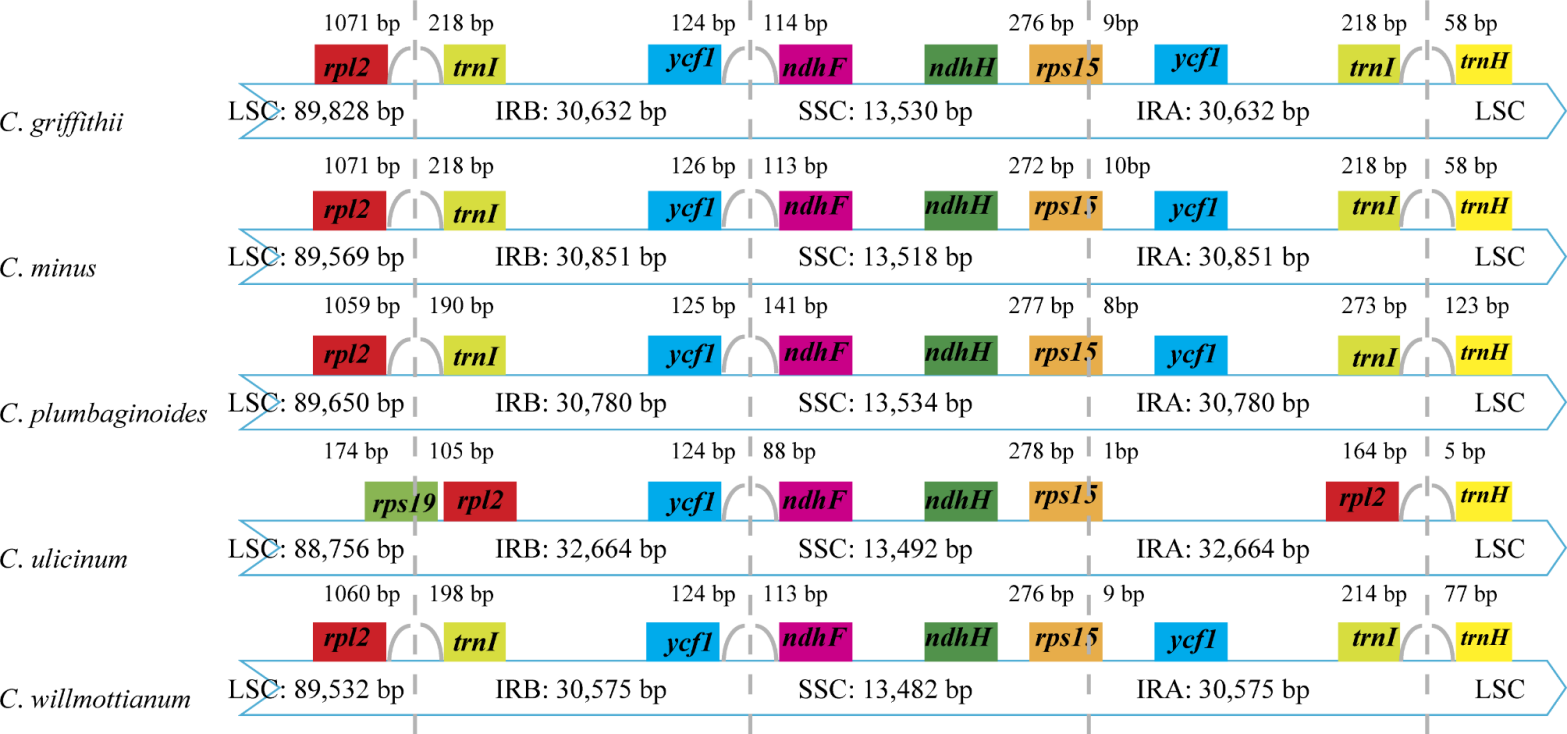
Comparison of the LSC, IR and SSC borders of five *Ceratostigma* plastomes

The differences between inverted repeats and single-copy (IR/SC) borders among the five *Cerotastigma* plastoms (GenBank accession numbers: OP954207 for *C*. *griffithii*, OP954208 for *C*. *minus*, OP954204 for *C*. *plumbaginoides*, OP921766 for *C*. *ulicinum*, OP967034 for *C*. *willmottianum*) were examined by comparative analyses (Table 1). As shown in Fig. 1, the plastome sequences were highly similar within the genus *Cerotastigma*. Except *C*. *ulicinum*, the IR/SC junctions were conserved in four other species. The LSC/IRB border in *C*. *ulicinum* plastome was positioned within *rps19* (with 105 bp located at IRB) and *rpl2* genes and the IRA/LSC border was *rpl2*/*trnH* genes. For the other *Cerotastigma* species, the LSC/IRB and IRA/LSC borders were *rpl2*/*trnI* genes and *trnI*/*trnH* genes, respectively. All species had the same IRB/SSC junctions, in which the *ycf1* genes and *ndhF* genes were 124–126 bp and 88–141 bp away from IRB/SSC borders. The *rps15* genes, crossing the SSC/IRA borders, were located at SSC and IRA regions with 272–278 bp and 1–10 bp, respectively.

**Fig. 2:**
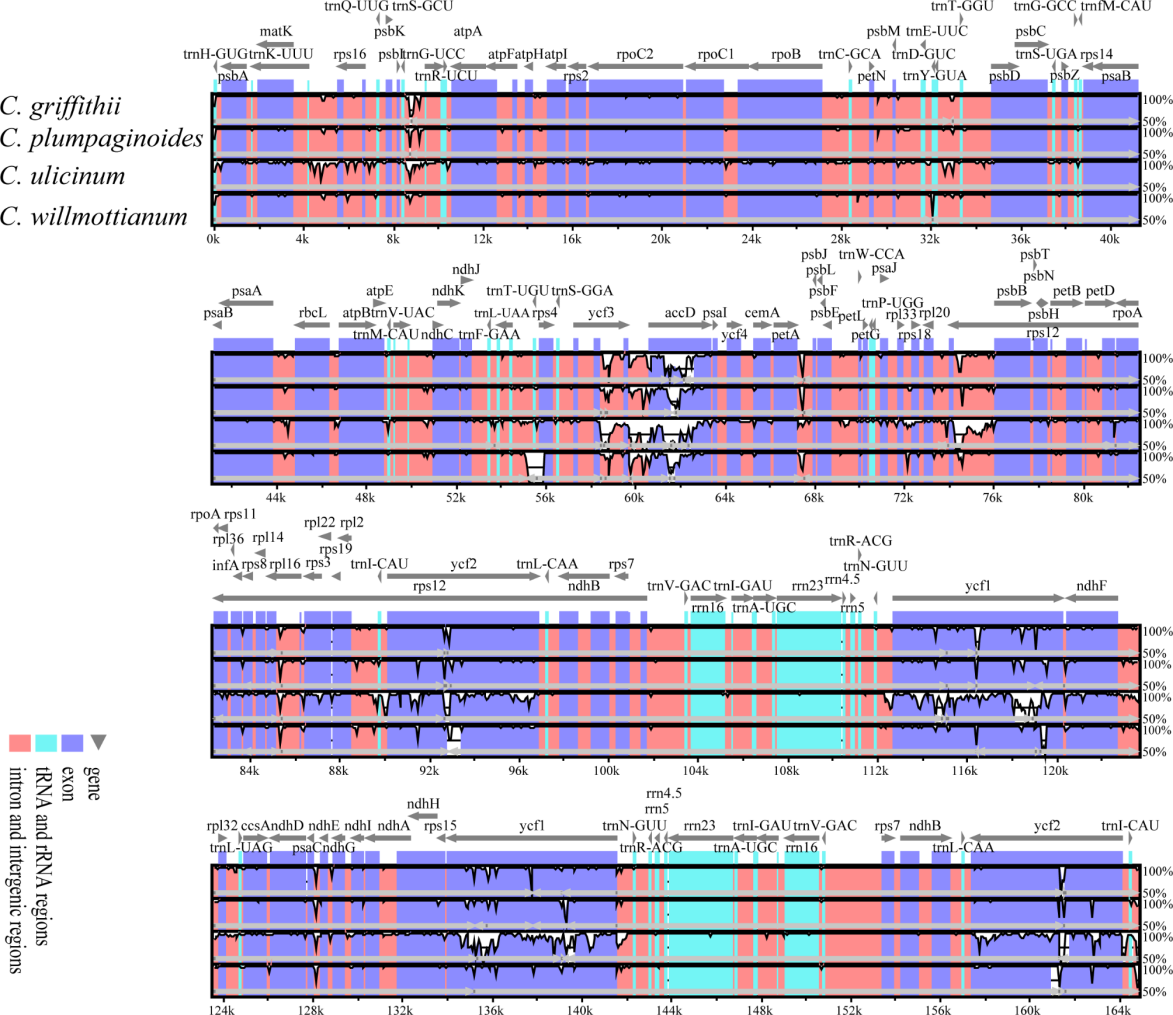
Sequence identity plots of five *Ceratostigma* plastomes. The plastome of *C*. *minus* was used as the reference genome. The horizontal axis represented the coordinates within the plastomes, and the vertical scale showed the percentage of identity within 50−100%. Grey arrows lines indicated gene orientation. Purple bars represented exons, blue bars represented tRNA and rRNA and red bars showed non-coding regions including introns and intergenic regions

**Fig. 3:**
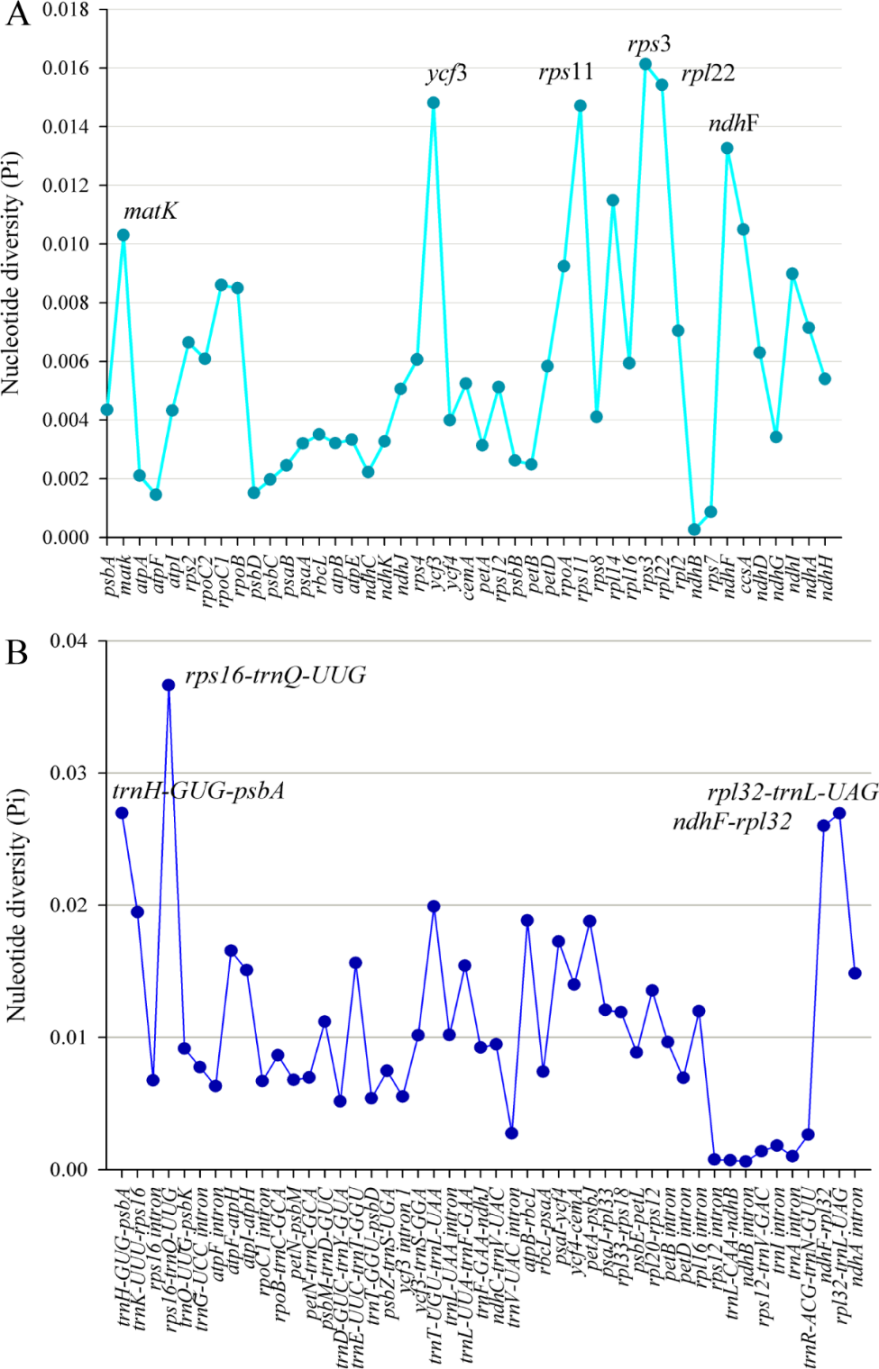
Nucleotide diversity (Pi) values of different regions in five plastomes in the genus *Ceratostigma*. **(A)** protein coding regions with Pi > 0.010 labeled with loci tags of genic names; **(B)** intron and intergenic regions with Pi > 0.020 labeled with loci tags of fragment names

The mVISTA results showed that the sequences in non-coding regions were more divergent than those in coding regions (Fig. 2). For the non-coding regions, high variation were found in *trnH*-*GUG*-*psbA*, *trnK*-*UUU*-*rps16*, *rps16*-*trnQ*-*UUG*, *atpF*-*atpH*, *atpI*-*atpH*, *trnE*-*UUC*-*trnT*-*GGU*, *trnT*-*UGU*-*trnL*-*UAA*, *trnL*-*UAA*-*trnF*-*GAA*, *atpB*-*rbcL*, *psaI*-*ycf4*, *petA*-*psbJ*, *ndhF*-*rpl32* and *rpl32*-*trnL*; and the main divergence for the coding regions were *matK*, *rpoC1*, *ycf3*, *rps11*, *rpl36*, *rps3*, *rps19*, *ndhF* and *rps15*. Moreover, we extracted all coding genes, intergenic and intronic loci from the five species, and calculated the nucleotide diversity (Pi) of loci with length longer than 350 bp. In the protein coding regions, the Pi values for each locus ranged from 0.00026 to 0.01613 and had an average value of 0.00603 (Table S2). Among these regions, six regions (*matK*, *ycf3*, *rps11*, *rps3*, *rpl22*, and *ndhF*) exhibited remarkably high variation with Pi > 0.01 (Fig. 3A, Table S2). The nucleotide diversity in intergenic and intronic regions ranged from 0.0006 to 0.03664, and showed higher average nucleotide diversity (0.0110) than that of coding regions. Four regions including *trnH*-*GUG*-*psbA*, *rps16*-*trnQ*-*UUG*, *ndhF*-*rpl32* and *rpl32*-*trnL* showing relatively high nucleotide diversity values larger than 0.02 were identified (Fig. 3B, Table S2).

### Analyses of repeat sequences

**Fig. 4:**
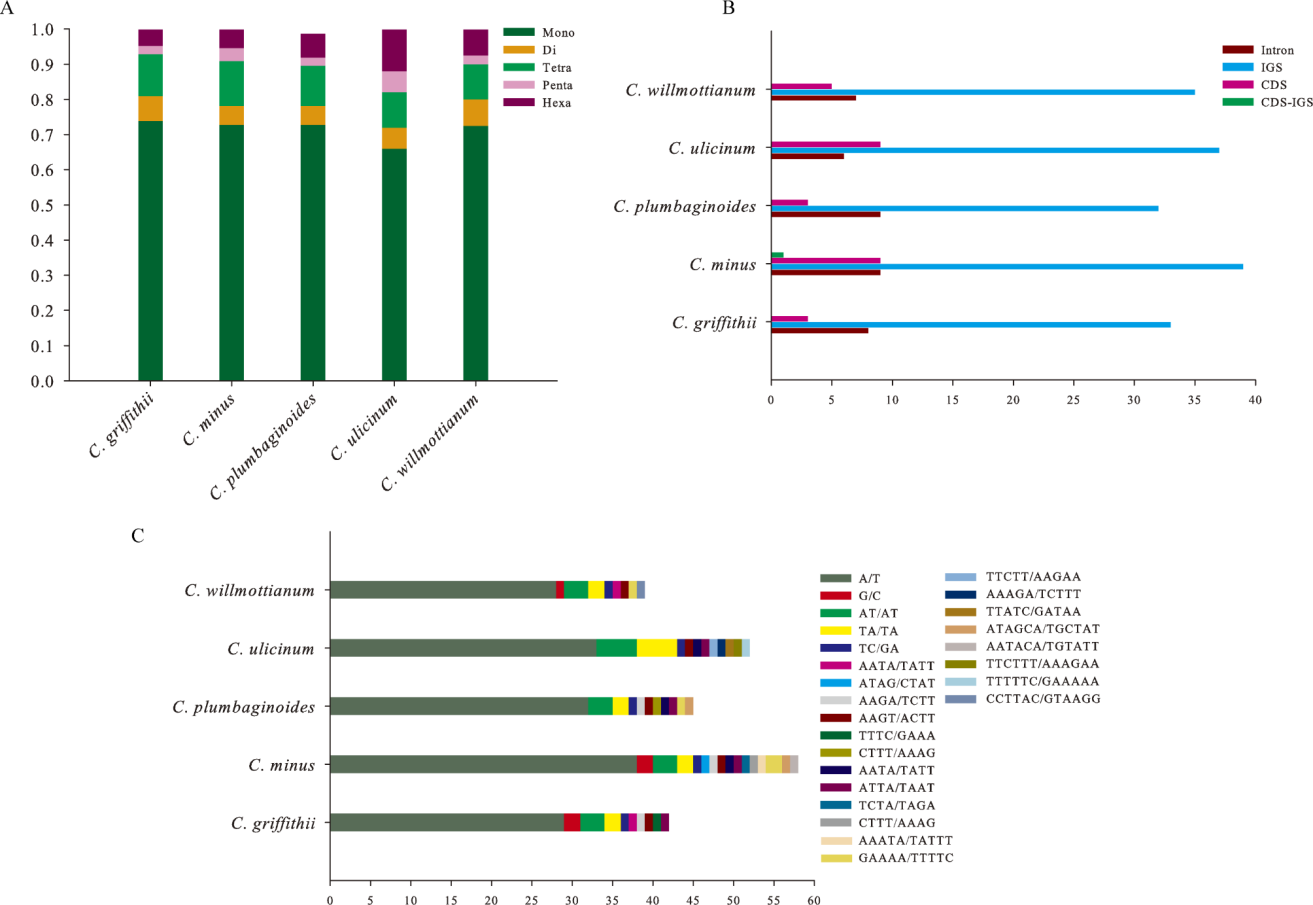
Analysis of simple sequence repeats (SSRs) in the five *Ceratostigma* plastomes. **(A)** the ratios of different types detected in the *Ceratostigma* plastomes within each species; **(B)** the number of SSRs in the introns, intergenic regions (IGS), protein-coding genes (CDS) and CDS-IGS (partly in CDS and partly in IGS); **(C)** the types and number of each identified SSR in the five *Ceratostigma* plastomes

**Fig. 5:**
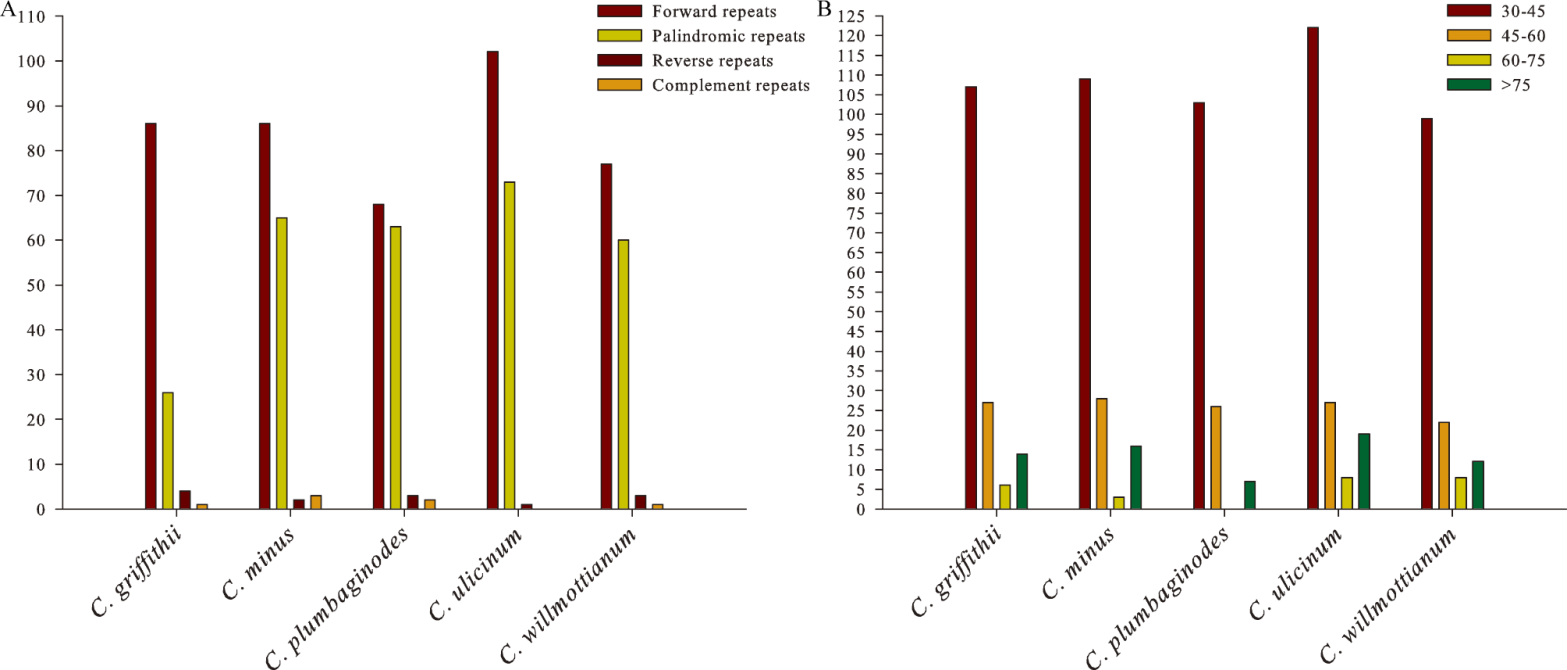
Long repeat sequences among the five *Ceratostigma* plastomes **(A)** total numbers of four repeat types (forward, palindromic, reverse and complement) detected; **(B)** numbers of long repeat sequences by length

A total of 231 simple sequence repeats (SSRs) were detected in the five *Cerotastigama* plastid genomes, in which *C*. *minus* contained the most SSRs, while *C*. *willmottianum* contained the least (Table S3). Specifically, five types of SSRs were identified (mononucleotide, dinucleotide, tetranucleotide, pentanucleotide and hexanucleotide), but trinucleotide repeats were not detected in all five species. Among these types, mononucleotide repeats were the most frequent in all five species, with ratios ranged from 0.6600 for *C*. *ulicinum* to 0.7381 for *C*. *griffithii*, followed by the ratios of tetranucleotide repeats ranging from 0.1000 for *C*. *ulicinum* to 0.1273 for *C*. *minus*, hexanucleotide repeats ranging from 0.0476 for *C*. *griffithii* to 0.1200 for *C*. *ulicinum*, dinucleotide repeats accounting for the lower ratios (0.0545–0.0750), and pentanucleotide repeats accounting for the lowest (0.0227–0.0600) (Fig. 4A, Table S3). The majority SSRs were distributed in the intergenic regions (IGS) (71.84%) and less in protein coding regions (15.92%) and the introns (11.84%) (Fig. 4B, Table S3). Among the mononucleotide repeats, A/T repeats accounted for the most part (96.97%) of all mononucleotide repeat types among the five plastid genomes. In the dinucleotide repeats, the AT/AT and TA/TA repeats were observed more frequently, with 43.75% and 40.63% of all dinucleotide repeats, respectively. In the tetranucleotide repeats, AAGT/TATT repeats were the most abundant type, with 22.73% of all tetranucleotide repeats types in the five plastid genomes (Fig. 4C, Table S3). Additionally, a total of 726 long non-overlapped repeats, including forward, palindromic, reverse, and complementary repeats with range 30–179 bp, were also detected in the five *Cerotastigama* plastid genomes using the online program REPuter. Collectively, repeat numbers and length varied from one species to another. *C*. *ulicinum* possessed the greatest number of repeats with the number of 176 and *C*. *griffithii* had the lowest (117). Most abundant were forward repeats ranging from 68 in *C*. *plumbaginodes* to 102 in *C*. *ulicinum*; complementary repeats were the least abundant repeats, ranging from zero in *C*. *ulicinum* to 3 in *C*. *minus* (Fig. 5A). As for the repeat length, the long repeats with 30–45 bp were found to be the most common, and those with length of 45–60 bp and > 75 bp were the second and third abundant types, respectively (Fig. 5B).

### Codon usage pattern and adaptive selection analyses

**Fig. 6:**
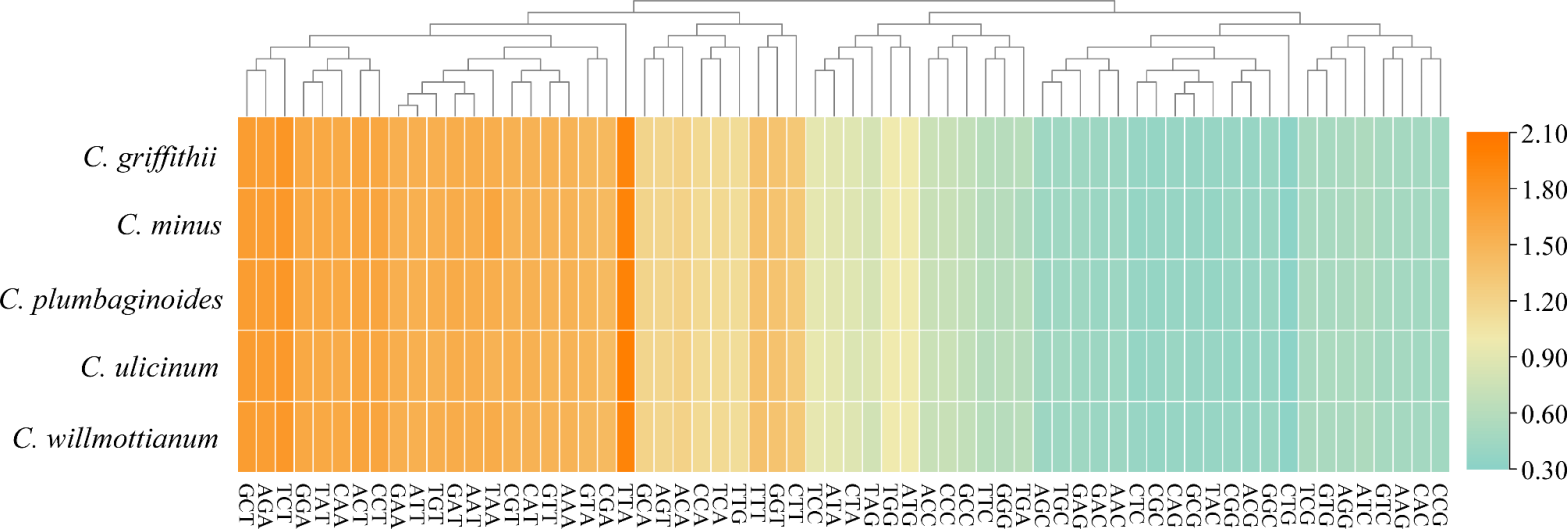
The codon usages of all protein-coding genes for five *Ceratostigma* plastomes. Orange colors indicate higher values of relative synonymous codon usage (RSCU) and blue color indicate lower RSCU values

The codon usages of the protein-coding genes in the plastomes from the five *Ceratostigma* species were analyzed. The total sequenced sizes of the protein-coding genes for codon analysis were 86,982−87,465 bp in the five *Ceratostigma* plastomes. The number of encoded codons ranged from 18,734 to 18,802 in plastomes. Leucines (Leu) was the most abundant amino acid with a frequency of 10.21−11.09%, followed by isoleucine (Ile) with a proportion of 7.75−8.47%, whereas cysteine (Cys) was coded by the least number of codons (203–226) (Table S4). The values of relative synonymous codon usage (RSCU) were shown in Fig. 6, of which 30 codons were used more frequently with RSCU > 1. Meanwhile, out of the above 30 codons, 29 codons possessed A/T at the third nucleotide positions except TTG. Conversely, most of the codons ended with G/C had RSCU values of less than one, indicating less commonly used in the five sequenced genes of the plastomes. The A/T bias at the third position of codons could also be inferred by the AT contents of codons. The mean values of AT content of the third codon positions were 74.7%. The usage of two codons ATG for methionine (Met) and TGG for tryptophan (Trp) had RSCU values of 1.00 and exhibit no codon bias (Fig. 6, Table S4).

**Fig. 7:**
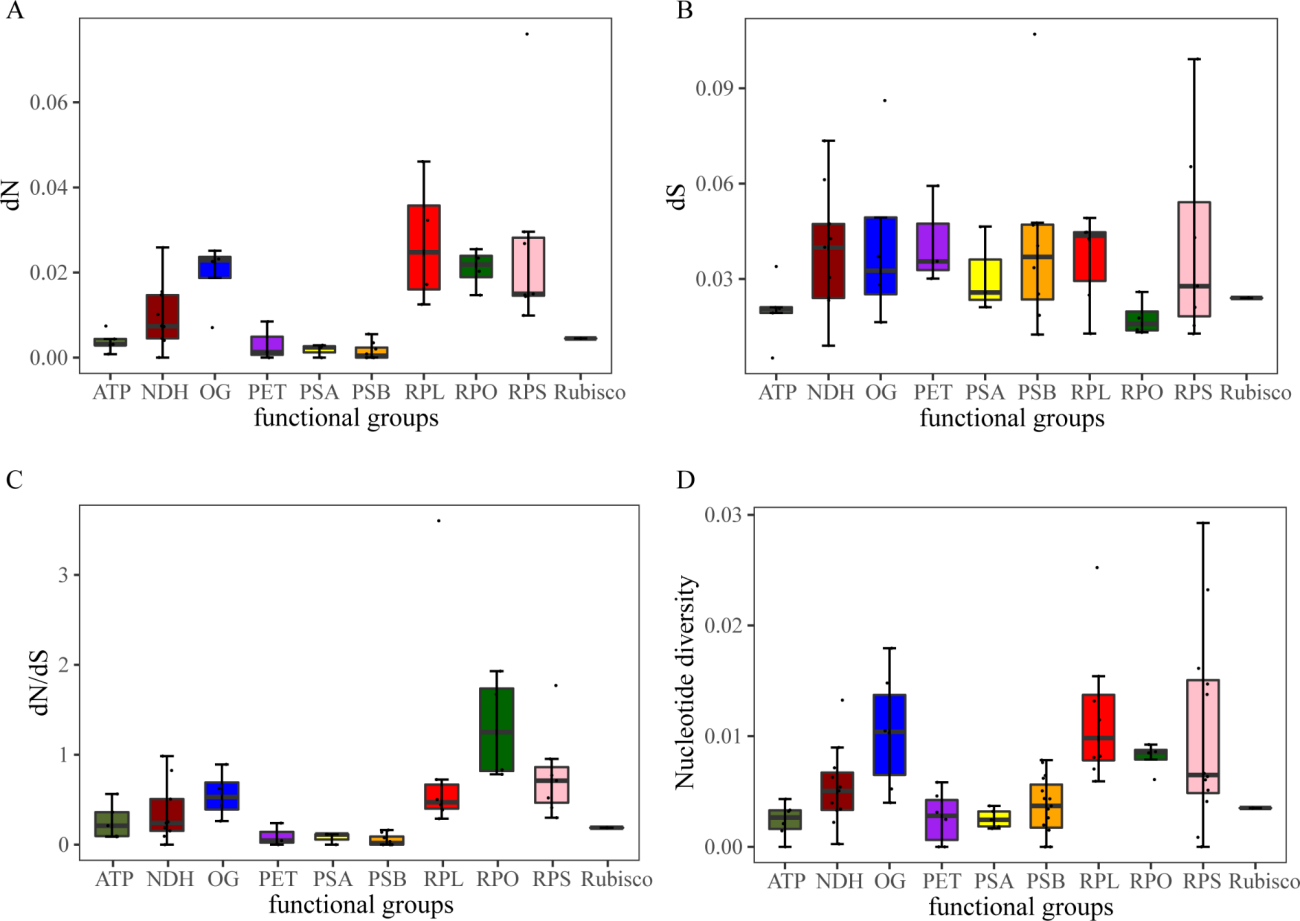
The results of selective pressure analysis in *Ceratostigma* plastomes. **(A)** estimates of non-synonymous nucleotide substitution (dN); **(B)** estimates of synonymous nucleotide substitution (dS); **(C)** estimates of dN/dS; **(D)** nucleotide diversity of different gene functional groups

According to the functional groups of the genes, the dN, dS and dN/dS were calculated to examine selective pressures and nucleotide diversity values of the different functional groups were also analyzed. Overall, the dN/dS ratios of most genes in the five *Ceratostigma* plastomes, were less than one, suggesting these genes went through purifying selection (Fig. 7, Table S5). When grouping genes into different functional groups, RPL (large subunit of ribosome) and RPO (DNA dependent RNA polymerase) had higher median dN and dN/dS values, respectively. Only four coding genes belonging to three different functional groups had dN/dS ratios > 1, such as *rpl22* belongings to the RPL functional group, *rpoA* and *rpoC1* belonging to the RPO functional group, and *rps15* belongings to the RPS (small subunit of ribosome) functional group. Among the four genes potentially experiencing positive selection, *rpoC1* and *rps15* had *P* values < 0.05 after the likelihood ratio test (LRT) under three site model comparisons. At the protein level for further analysis based on the Bayes empirical Bayes (BEB) approach [30], four and two positive amino acids sites for *rpoC1* and *rps15* genes were identified as under positive selection, respectively (Fig. 7, Table S5). Accordingly, genes within RPO, RPL and RPS functional groups possessed relatively high median nucleotide diversities than those of other functional groups, perhaps indicating them as faster evolving genes (Fig. 7, Table S5).

### Phylogenetic inferences

**Fig. 8:**
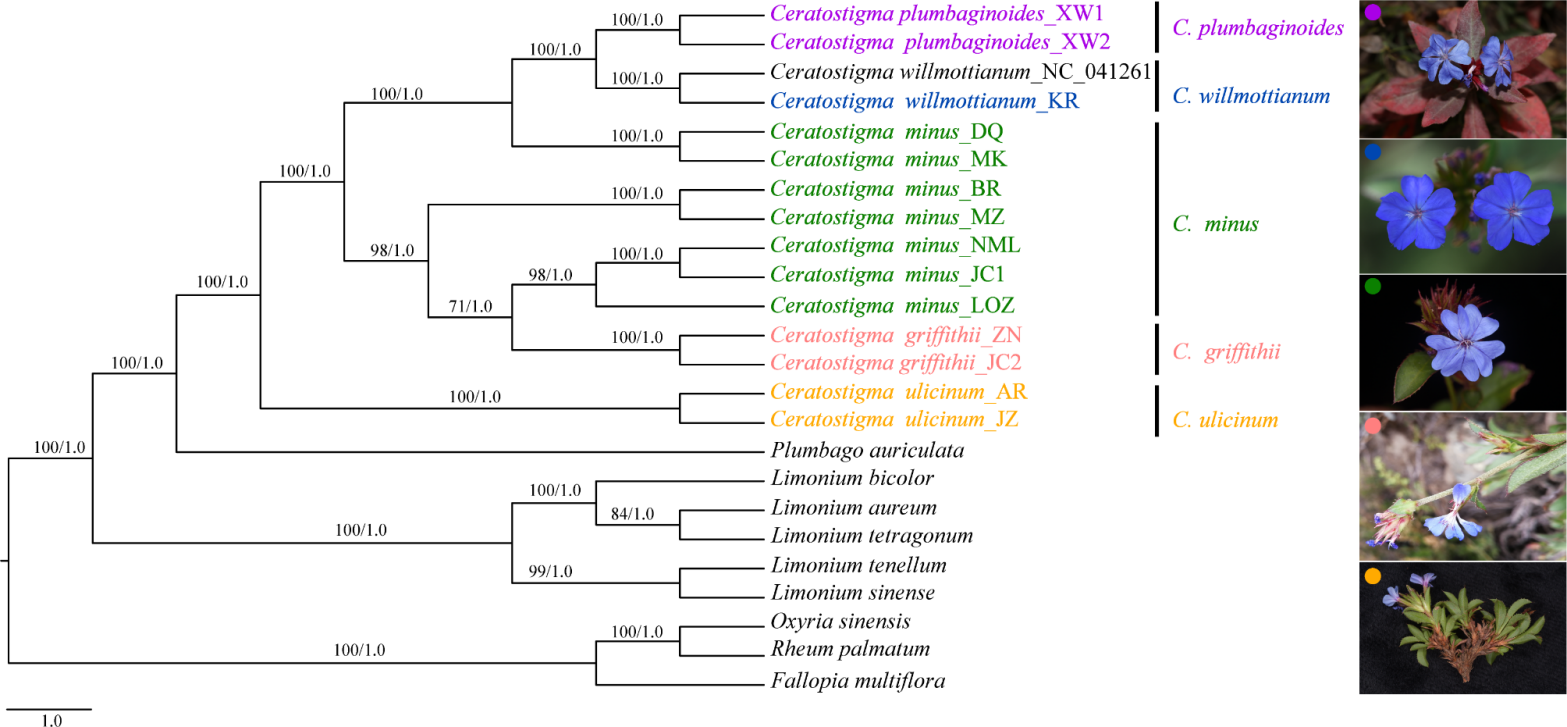
The phylogenetic trees of all accessions based on chloroplast genome sequences. The number above lines indicates bootstrap values for maximum likelihood (ML) and posterior probabilities for Bayesian inference (BI) analyses of the phylogenetic analysis for each clade. The five species are shown by different colors that are used in the sampling map (Fig. S5). A picture of species is shown on the right, with each pie color corresponding to that of each species in the tree

Phylogenetic positions and interspecific relationships of *Cerotastigma*, together with five *Limonium* and one *Plumbago* species within Plumbaginaceae were analyzed (Table S6, Table S7). Based on the whole plastid genomes with half gap positions allowed, the optimal base substitution model calculated by jModeltest was GTR + I + G under the rule of AIC (Akaike information criterion). Two approaches maximum likelihood (ML) and Bayesian inference (BI) produced congruent tree topologies, in which the monophyly of the genus *Ceratostigma* was strongly supported in both cases (bootstrap value (BS) = 100%, posterior probability (PP) = 1.0), closely related with *Plumbago* (Fig. 8). The infragenenic phylogeny was well resolved and most nodes were strongly supported. Only one node, including *C*. *minus*_NML, *C*. *minus*_JC1, *C*. *minus*_LOZ, *C*. *griffithii*_ZN and *C*. *griffithii*_JC2, was not strongly recovered (BS = 71%, PP = 1.00). Four of the five species that had more than one accession were resolved as reciprocally monophyletic, with the exception of *C*. *minus*, for which samples from Hengduan Mountains (*C*. *minus*_DQ and *C*. *minus*_MK) and QTP (*C*. *minus*_BR, *C*. *minus*_MZ, *C*. *minus*_NML, *C*. *minus*_JC1 and *C*. *minus*_LOZ) formed distinct clades (Fig. 8). *C*. *ulicinum* occupied an isolated position and was sister to all other species with full support. *C*. *griffithii* was retrieved as sister to a clade containing three accessions of *C*. *minus* sampled from Shigatse (NML) and Lhoka regions (JC1 and LOZ) in the QTP, respectively; *C*. *plumbaginoides* distributed in North China were resolved as sister to *C*. *willmottianum*. For genus *Limonium*, *L*. *bicolor* clustered with *L*. *aureum and L*. *tetragonum*, and formed a clade with high support (BB = 100%, PP = 1.0); the other clade was composed of *L*. *sinensis* and *L*. *tenellum* with more close relationship. The phylogenetic trees based on the whole plastid genomes with all or none gap positions allowed, using base substitution model GTR + I + G and TVM + I + G showed similar topologies, and the species from the same genus clustered together (Fig. S2). Moreover, the results from the nrDNA dataset produced almost congruent relationships with that of the plastome dataset, other than a few discrepancies in phylogenetic placement of some individuals (Fig. S3). The differences between the two phylogenies were mostly restricted to areas of poor support. For instance, in the nrDNA phylogeny, *C*. *griffithii*_JC2 was more closely related to *C*. *minus*_DQ *and C*. *minus*_MK with weak support (BS = 40%, PP = 0.6418), whereas in the plastome phylogeny, two species of *C*. *griffithii* formed a clade and was resolved as sister to one clade of *C*. *minus* including *C*. *minus*_LOZ, *C*. *minus*_JC1 and *C*. *minus*_NML (Fig. 8). Similarly, the systematic positions of several *C*. *minus* individuals were also not well resolved and varied in ML and BI analyses. The phylogeny inferred from the combined datasets of nrDNA and plastomes (Fig. S4) largely resembled the tree topology of plastome data, and four species were recovered as monophyletic with high support, but individuals of *C*. *minus* still failed to form a unique cluster, which showed a trend of geographical clustering visible in the ML and BI trees. When compared with each other, the plastome phylogeny was better supported than that of nrDNA, and resolution and node support values were significantly improved by the combined dataset.

## Discussion

### Plastome structure and sequence variation

The plastome structure is generally conserved in most angiosperm and comparative analyses have been used to examine the differences in plastome evolution. In this study, 14 *Cerotastigma* plastomes were generated. We found that the plastomes in *Cerotastigma* are highly conserved, exhibiting little differences in terms of gene number (127–128) and IR/SC borders. Comparatively, genome size among the five species and within different individuals of the same species showed some variations (Table 1), which have been reported in other species, such as *Dipelta floribunda* and *Dipelta yunnanensis* [31] and *Colligonum* [22]. These differences may be attributed to the extraction or expansion in Inverted Repeats regions, which were regarded as a common evolutionary phenomenon in plastome evolution [32]. The detailed comparison of the five IR/SC junctions of the *Cerotastigma* plastomes showed similar characteristics but expansion in IR region with shifts in gene positions of *C. ulicinum*, resulting in its longest plastome length (Fig. 2). The changes in position of IR/SC border were also observed in plastid genomes in other species belonging to Plumbaginaceae, due to the duplication to the part of the *ycf1* gene [33]. Additionally, the GC content of *Cerotastigma* (37.3–37.7%) was comparable to that of *Limonium* (36.7–37.1%) [23] and *Plumbago* (37.1%) [15] published in Plumbaginaceae (Table 1). The IR regions had higher GC content (40.7–41.4%) than those of LSC region (35.6–36.0%) and SSC region (31.9–32.2%). These results reported here have similarity among other species, most likely due to the high GC content in rRNA genes [34, 35].

The divergent hotspot regions dispersed throughout the plastomes could provide variable information and contribute to further researches exploring angiosperm relationships and relating to population genetics [36, 37]. In accordance with most previous studies on the plastomes of angiosperms, we also found that the nucleotide sequence diversity of the non-coding regions was higher than that of the coding regions (Fig. 3). Unlike previous markers such as *matK*, *rbcL* and *trnL*-*F* employed in Plumbaginaceae [13, 23], more highly variable regions (*ycf3*, *rps11*, *rps3*, *rpl22*, and *ndhF*, Pi > 0.01) were identified in the coding regions; four intergenic regions including *trnH*-*GUG*-*psbA*, *rps16*-*trnQ*-*UUG*, *ndhF*-*rpl32* and *rpl32*-*trnL* had high nucleotide diversity values (Pi > 0.02). These regions could serve as potential molecular markers for further phylogenetic and population genetics studies.

Besides genes or intergenic fragments, plastid simple sequence repeats (cpSSRs) markers possess unique and important variations, and are also potential tools to investigate population genetic variation and phylogeographic patterns [38, 39]. A total of 40 (*C*. *willmottianum*) to 55 (*C*. *minus*) cpSSRs were found and vastly distributed in the intergenic region (IGS) of five species (Fig. 4). Most of the SSRs types are mononucleotide repeats (A/T), whereas di- or trinucleotide repeats are rare. This result was similar to the repeat characteristics of other reported gymnosperms and angiosperm plastoms [21, 40–42] and it is suggested that more abundant A/T motifs could keep a more stable framework compared to polyC and polyG [43]. In addition to short sequence repeats, abundant long repeat sequences were also identified, which play an important role in promoting plastome rearrangement and sequence divergence [44–46]. Among the five congeneric species, repeat numbers varied from one species to another (117 for *C*. *griffithii* and 176 for *C*. *ulicinum*) (Fig. 5). The type of 30–45 bp repeat accounted for the largest number, and this observation is congruent with the results of unrearranged plastomes [37, 47].

We further analyzed the codon usage and relative synonymous codon usage frequency (RSCU) in the five *Cerotastigma* plastomes. In this investigation, the more frequent amino acid was leucine, followed by cysteine identified as a rarest amino acid. The findings were comparable to those observed in *Limonium* [23]. In the 64 mutant codons including three stop codons, 32 codons possessed A/T at the third nucleotide positions and 30 of which showed RSCU values larger than one, indicating the higher content of A/T used in the codons and especially at the third codon position (Fig. 6). In general, codons with a high AT content are used more often in plastomes [48]. Although several exceptions were also reported such as in Geraniaceae with high GC content of protein-coding genes [49], the same tendencies of favored codon usage pattern followed for many other plastomes, which might be correlated with the high proportion of A/T in plastid genomes or induced by adaptation evolution of the plastid genomes [49, 50].

### Plastome evolution

Furthermore, to investigate the possible gemonic evolution, we estimated the selective pressures of common protein-coding genes in *Cerotastigma* plastomes based on the useful measuring selection pressure parameter (dN/dS) at the protein level. In most genes, synonymous nucleotide substitution generally occurred more frequently than non-synonymous nucleotide substitution ones, and consequently, most genes evolved under purifying selection or displayed neutral evolution [51]. We found that most of the dN/dS values for the genes were less than one, representing that the genes in *Cerotastigma* plastomes were subjected to purifying selection. When grouping genes into different functional groups, the functional groups RPO and RPS had higher median dN and dN/dS values, suggesting that accelerated substitution rates were detected in genes involved in transcription and translation when compared with those participated in photosynthesis systems (Table S1). These results were similar to what have been found in some land and aquatic plants [52, 53]. In our comparisons, specifically, dN/dS values of two genes *rpoC1* and *rps15* were larger than one and positive selection sites were identified (Fig. 7). The gene *rpoC1* encodes DNA dependent RNA polymerase involving in transcription and *rps15* belongs to ribosome small subunit gene encoding ribosomal protein, and both play important roles in transcription and translation. Such functional genes with signature of positive selections were also detected in *Rheum* experienced rapid radiations in the QTP [21], and in grasses dominated as savannas belonging to several sub-families such as Panicoideae and Arundinoideae [54–56]. It is proposed that *Cerotastigma* probably have a tropical origin [2], and according to our field investigation, some of five species are distributed parapatrically and possess similar dry habitats in the QTP*sl* [4, 57]. Therefore, the narrow spectrum of candidate gene functional classes affected may on the one hand, reflect the typically conservative plastid genome across most angiosperms; on the other hand, might mirror adaptation of species in the genus *Cerotastigma* to similar but different environments with gradually increased elevations and longitude [58, 59].

### Phylogenetic relationships

The availability of plastid genome sequence has provided many new insights into gymnosperms and angiosperm phylogenetic relationships at the ordinal, familial, tribal and lower levels [16, 18, 20, 60]. In Plumbaginaceae, the plastid genomes remained very limited, and only several species were sequenced. In this study, we obtained 14 plastid genomes of five *Cerotastigma* species distributed in China and multiple individuals from different populations per species were included in our analyses. By incorporating the sequenced plastomes in Plumbaginaceae, we were able to gain some insights into the interspecific relationships of the small genus of *Cerotastigma*. Our result suggested that *Cerotastigma* was monophyletic and closely related to *Plumbago*, as identified in phylogenomic works of Caryophyllales [15] and molecular phylogenetic results of Plumbaginaceae [13]. Within the genus *Cerotastigma*, the available evidence suggested that *C*. *ulicinum* was monophyletic with high support and formed a clade sister to the other clades composed of four species (Fig. 8). *C*. *ulicinum* is a cushion shrub, and has the most restricted distribution only in the South Tibet of China. The species has unique morphological characteristics, i.e., rigid and linear to almost needlelike bud scales [1], which could be explicitly distinguished from other species within *Cerotastigma*. Thus, in accordance with the morphological differentiation, our data supported the species was highly genetically differentiated from other species across the sampled distribution.

Additionally, the subshrub *C*. *willmottianum* with main extant distribution in Hengduan Mountains was retrieved as sister to the herb *C*. *plumbaginoides*, whose life form is not dominant in arid areas in the QTP*sl* and distribution range is more northward. *C*. *griffithii* and *C*. *minus* are morphologically more similar and also have parapatric distributions in the QTP*sl*, and this explains their close relationship in the plastomic phylogeny. In particular, currently, *C*. *minus* was not recognized as a monophyletic group, but had two distinctly main lineages divided by Mekong-Salween Divide (MSD) [61]. These results indicate that groups of species within *Ceratostigma* tend to exhibit a high degree of geographic endemicity corresponding to their clade affiliation, in which samples from different populations per species seem to be clustered by geography other than species. Taking the distribution range into consideration, it is perhaps reasonable to pinpoint that geographic isolation is one of the major precursors to promote interspecific/intraspecific genetic divergence of *Ceratostigma*. Mekong-Salween Divide composed by large mountains like Nushan Mountains has been invoked to explain genetic discontinuities or cryptic speciation in a wide range of different species in the Himalaya and Hengduan Mountains regions, such as *Sinopodophyllum hexandrum* [62], *Taxus wallichiana* [63] and *Marmoritis complanatum* from the subnival belt [64]. Yet such geographical structure of *C*. *minus* may also reflect genealogical processes including hybridization and introgression [65]. Especially when hybridization and subsequent backcrossing occurred, the plastome of one species might be captured by the other [66], the phenomenon of plastid capture could occur frequently with closely related species with sympatric distribution and reproductive compatibility [67–69]. Within *Ceratostigma*, interspecific hybridization has been observed and inferred, for example, morphological intermediates of leaves between *C*. *minus* and *C*. *griffithii* are described in Yunnan and adjacent areas in Sichuan province [1], suggesting hybridization or introgression may occur among at least some populations of these species. When considering the parapatric distribution of *C*. *griffithii* and *C*. *minus* such as in Lhoka regions in Tibet, the possibility of gene flow between them may not be completely excluded. However, we also noticed that the plastome-based phylogeny of the *Ceratostigma* represents one aspect of the overall evolutionary history of the group. It is essential to compare plastid phylogenies against those from nuclear genome to assess the influence of multiple processes on the phylogenetic relationships.

Unfortunately, the results inferred from the nrDNA dataset could not provide solid resolution for the phylogenetic relationships of *Ceratostigma*, perhaps due to its short length and not enough parsimony-informative characters. Especially, the systematic positions of individuals of *C*. *minus* and *C*. *griffithii* varied from those of plastome and combined datasets, and similar results were also found in other phylogenetic studies [70, 71]. This phenomenon could be better explained by different important determinants including incomplete lineage sorting, hybridization/introgression and other genetic processes [72, 73]. Currently, except *C*. *willmottianum*, more than one individual of the species were sampled and included in the phylogenetic reconstruction, and it should be noticed that genome skimming data discards a large portion of the nuclear genome, thus may limit the power of discrimination at, or below, the species level [74]. Moreover, gene flow could leave similar traces in the genome to those created by incomplete lineage sorting [75], consequently, based on available data, pinpointing cases due to either phenomenon is difficult. It is therefore important to use more phylogenetic markers from genome for reconstruction of true relationships among the species. Further studies involving population genomic data and analyzed approaches could help to better elucidate their evolutionary histories.

## Methods

### Plant material sampling and genomic DNA extraction

In total, we included 14 samples representing all five extant species of *Ceratostigma* distributed in China (Fig. S5, Table S6). Among the five species, four had more than one individual sampled except *C. willmottianum*, whose plastome had been reported. Fresh leaves were collected from the field in Yunnan, Tibet and Henan, and then dried in silica-gel immediately. Because the *Ceratostigma* species we collected from field were currently not protected species, no permission was required during the sampling process. The formal identification of *Ceratostigma* species in this study was undertaken by Yujuan Zhao with help provided by Prof. Heng Li (Kunming Institute of Botany, Chinese Academy of Sciences), the author of the study of areas of the genus *Ceratostigma* [2]. Voucher specimens were deposited at the herbarium of Kunming Institute of Botany, Chinese Academy of Sciences under voucher specimens numbers GX008, GX025-026, GX047, ZYJ001, ZYJ006-007, ZYJ013-015, ZYJ034, ZYJ044 and XW001 (Table S6). The genomic DNA was extracted from silica-dried leaves using a modified CTAB method [76].

### Plastid genome sequencing, assembly and annotation

In total, 250–500 ng DNA from each sample was used to prepare libraries. Sequencing was conducted on an Illumina HiSeq X Ten platform with a paired-end of 150 bp reads. We checked the sequencing quality of raw reads with FASTQC (http://www.bioinformatics.babraham.ac.uk/projects/fastqc) [77], which were filtered by removing reads with low sequencing qualities such as duplicate reads and adapter-contaminated reads. The obtained clean reads were then assembled de novo using GetOrganelle tookit [78], and the main parameters were adjusted according to the assembled results. The plastid genome sequence of *C*. *willmottianum* (MK397862) was utilized as reference [15]. Plastid genomes were annotated using the online program GeSeq [79] and adjusted manually in Geneious v.9.0.1 [80] by comparing these with previously published plastomes of Plumbaginaceae, and the physical maps were drawn and illustrated using Organellar Genome Draw (OGDRAW) (https://chlorobox.mpimp-golm.mpg.de/OGDraw.html) [81]. The final annotated plastomes were deposited in GenBank with accessions listed in Table 1.

### Genome comparison and divergent hotspot identification analysis

The plastomes structure variation of the five species was examined by comparing the positions of SC/IR junctions and their adjacent genes to assess the expansion/contraction of the IR regions. All five complete plastomes were also aligned and compared with mVISTA in shuffle-LAGAN model [82], using *C*. *minus* as a reference. In order to identify regions of high genetic divergence between *Cerotastigma* species that could be potentially be informative for phylogenetic studies within this genus, the coding genes and intergenic regions (including introns) longer than 350 bp referred to Zhang et al. [83] were extracted from the plastomes using R scripts and the nucleotide diversities (Pi) within these regions were calculated in DnaSP v5.0 [84].

### Codon usage and repeat sequences analyses

The protein-coding genes were extracted from the five *Ceratostigma* plastomes in Geneious v9.0.1 [80]. The parameter of codon usage and relative synonymous codon usage (RSCU) were estimated in CodonW v1.4.2 program [85]. When RSCU values are larger than 1, these codons are more often used than expected. Simple sequence repeats (SSRs) were identified in these plastome sequences using GMATA v2.3 [86], with minimum repeat numbers of 10 for mononucleotide (momo-) repeats, 5 for dinucleotide (di-) repeats and trinucleotide (tri-) repeats, and 3 for tetranucleotide (tetra-), pentanucleotide (penta-), and hexanucleotide (hexa-) repeats, respectively. Moreover, REPuter program [87] was employed to identify long repeats including forward, palindromic, reverse, and complementary repeats. The parameters were set as follows: Hamming distance of 3, a repeat identity of more than 90% and minimum repeat size of 30 bp. All overlapping repeat sequences within the plastomes were not considered in the statistical analyses.

### Selective pressure analyses

To detect the protein-coding genes under selection in the five *Ceratostigma* species, the ratio (ω) of non-synonymous (dN) to synonymous (dS) nucleotide substitution rates were calculated utilizing the CodeML algorithm implemented in PAML v4.9j [88]. In these analyses, each single-copy CDS was extracted and aligned separately using MUSCLE Alignment in Geneious v9.0.1 [80] and the stop codons were deleted. The codon frequency was determined by the F3 × 4 model. Positive selection was determined when the value of ω was larger than 1. Then we further compared three pairs of site-specific model (M0 vs. M3, M1 vs. M2 and M7 vs. M8) to analyze the significances, in which the likelihood ratio test (LRT) values under the different model was compared [30] and the *P* values were calculated using Chi-square [89] to confirm the quality of the sets.

### Phylogenetic analysis

Phylogenetic relationships within Plumbaginaceae were reconstructed using the plastomes of species sampled in our comparative analysis and other species with plastomes publicly available in NCBI. We downloaded three plastomes of the sister family Polygonaceae (*Oxyria sinensis*, GenBank accession number: MK397882; *Rheum palmatum*, GenBank accession number: NC_027728; *Fallopia multiflora*, GenBank accession number: MK330002) as outgroups (Table S7) [15]. Multiple alignments of these matrices were made using MAFFT v.7.310 with default settings [90]. To evaluate the phylogenetic effects of character inclusion/exclusion and to minimize systematic error due to poor alignment, the conserved loci were selected by Gblocks v0.91b [91] with three different gap positions treated methods (none, half and all) and other parameters were set as default. In addition, two more datasets were analyzed to explore the phylogenetic relationships within *Ceratostigma*, one using the nuclear ribosomal DNA (nrDNA) data and the other with all plastome and nrDNA data combined. Reads containing nrDNA sequences from the genome skimming dataset of all *Ceratostigma* samples were collected and assembled using the same method as assembling plastomes. Regions composed of 18 S, ITS1 (internal transcribed spacer), 5.8 S, ITS2 and 26 S were well aligned and used for phylogenetic analysis. Three nrDNA sequences (GenBank accession numbers: MZ366771, MZ366779 and MZ366785) of *Opuntia* belonging to Caryophyllales were downloaded from NCBI to be used as outgroups (Table S7). Maximum likelihood (ML) and Bayesian inference (BI) methods were used to infer the phylogenetic relationships. The best substitution model determined by jModeltest v1.5 [92] were selected in BI analyses, and Marko chain Monte Carlo (MCMC) algorithm was run for one million generations, with one tree sampled every 1000 generations implemented in MrBayes v3.2 [93]. GTR + G model with 1000 rapid bootstrap replications for each matrix were set for ML analyses performed with RAxML v8.3 [94].

## Conclusions

In conclusion, we determined the complete plastome sequences of five species including 14 samples from different populations in China. The comparative analysis of these plastomes exhibited high similarities in terms of the overall structure, SSR, long repeat sequence and codon usage, but expansion in IR region with shifts in gene positions of *C. ulicinum* was detected. The highly polymorphic regions identified in the current study might be suitable for the phylogenetic analysis and resolving taxonomic discrepancies at the genus level. Moreover, our data resolves the phylogenetic relationships of *Cerotastigma* and establishes monophyly of the genus *Cerotastigma*. However, interspecific delimitation and relationships of four species were well resolved except *C*. *minus*, which was shown to be non-monophyletic, indicating that lineage genetic divergence perhaps was promoted by geographic barriers in Himalaya and Hengduan Mountains regions, but hybridization or introgression may not be excluded. These findings represent the first important step in elucidating plastome evolution and phylogenetic relationship in this widespread distribution genus *Cerotastigma* in the QTP*sl*. In the future, additional evidence from genomic data is needed to comprehensively uncover the evolutionary history of *Cerotastigma*.

## Electronic supplementary material

Below is the link to the electronic supplementary material.

Supplementary Material 1

Supplementary Material 2

Supplementary Material 3

Supplementary Material 4

Supplementary Material 5

Supplementary Material 6

Supplementary Material 7

Supplementary Material 8

Supplementary Material 9

Supplementary Material 10

Supplementary Material 11

Supplementary Material 12

Supplementary Material 13

## Data Availability

All annotated chloroplast sequences data in this study have been submitted to NCBI (https://www.ncbi.nlm.nih.gov/) with accession numbers OP954204-OP954210, OP967032-OP967036 and OP921765-OP921766 shown in Table 1. Other chloroplast genomes for phylogenetic analysis can be obtained from NCBI and their accession numbers are listed in Table S7. All voucher specimens were deposited in the herbarium of Kunming Institute of Botany, Chinese Academy of Sciences.
