# Supplementary figures and images for: Characteristics of plastid genomes in the genus Ceratostigma inhabiting arid habitats in China and their phylogenomic implications
Source: BMC Plant Biol. 2023 Jun 7;23:303. doi: 10.1186/s12870-023-04323-7 (PMC10245475; doi:10.1186/s12870-023-04323-7)

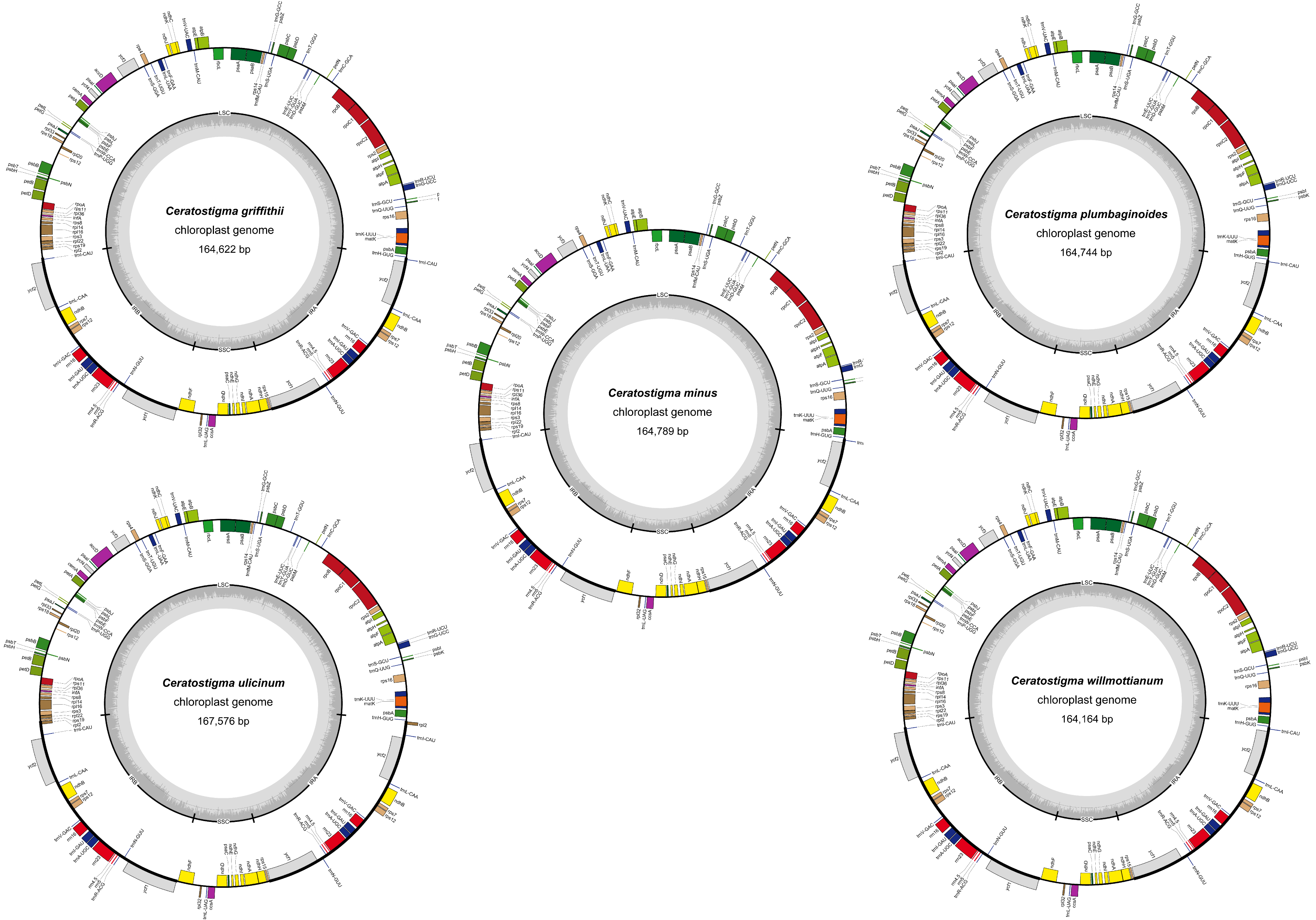

Supplement: Supplementary file 1 — Supplementary Material 1 [file 12870_2023_4323_MOESM1_ESM.tif]

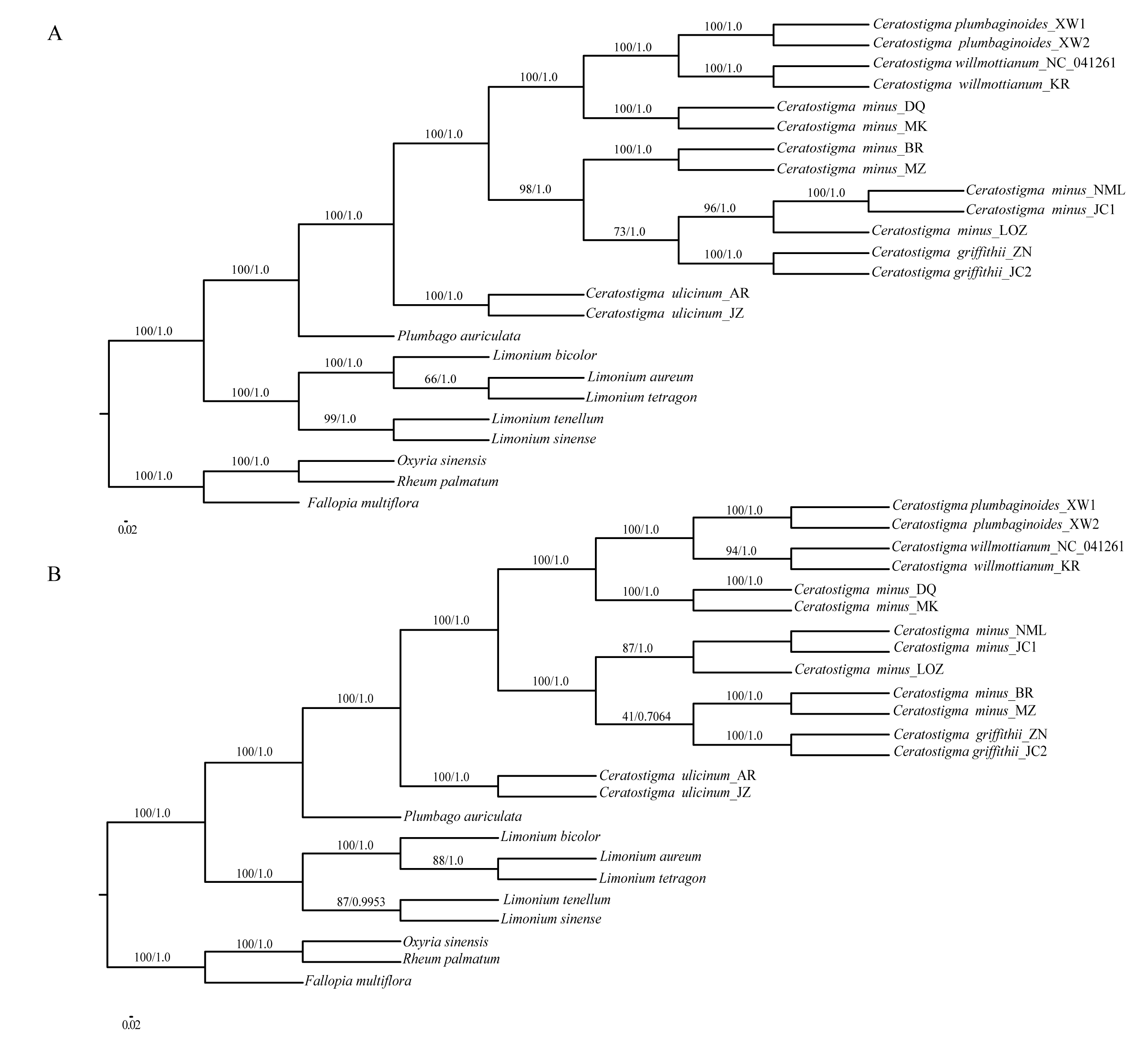

Supplement: Supplementary file 2 — Supplementary Material 2 [file 12870_2023_4323_MOESM2_ESM.tif]

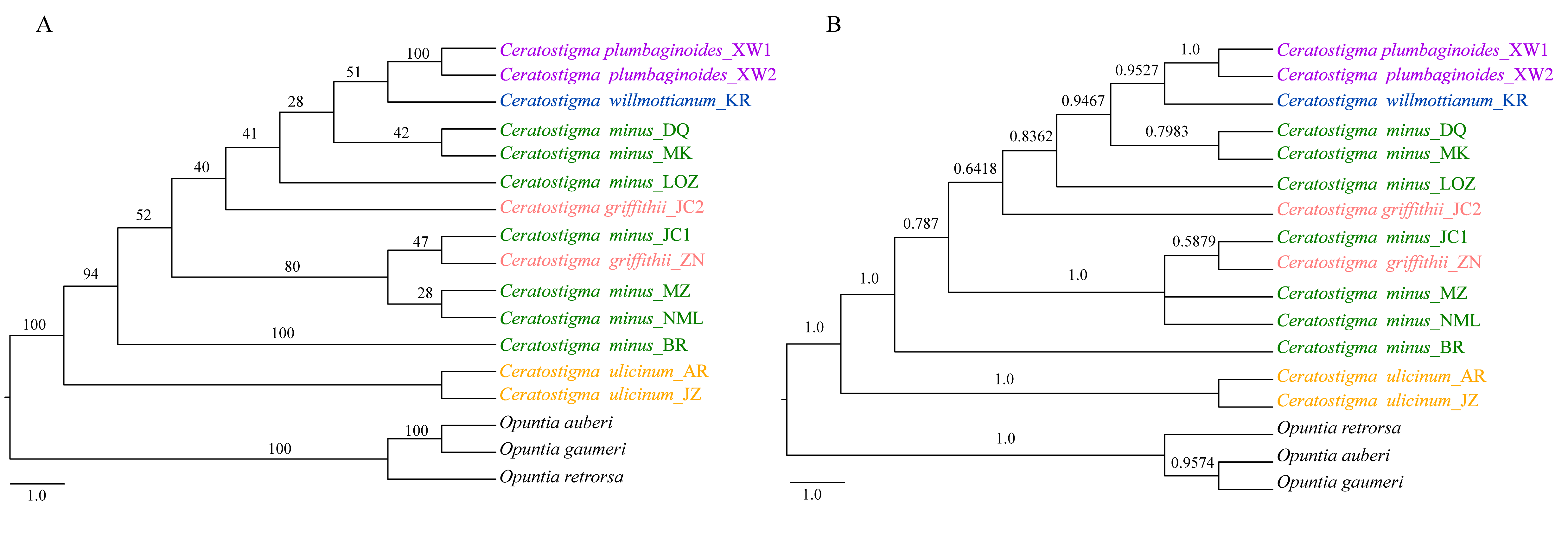

Supplement: Supplementary file 3 — Supplementary Material 3 [file 12870_2023_4323_MOESM3_ESM.tif]

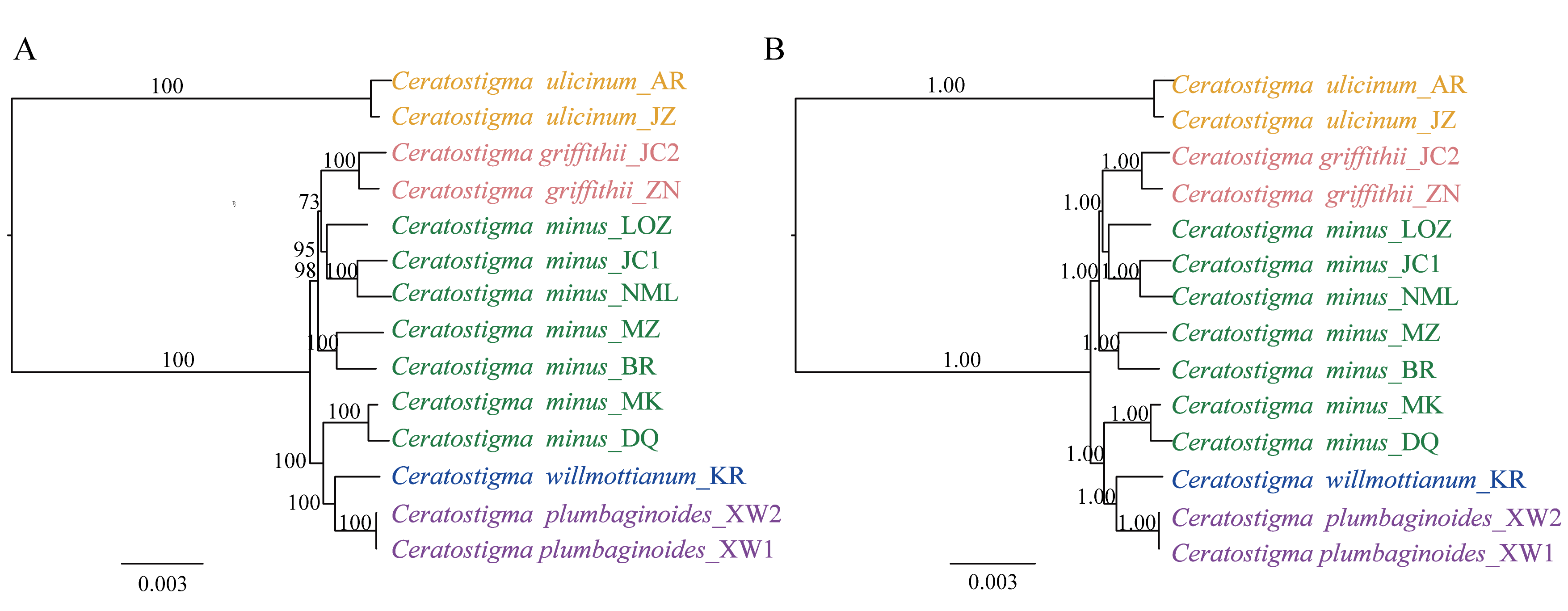

Supplement: Supplementary file 4 — Supplementary Material 4 [file 12870_2023_4323_MOESM4_ESM.tif]

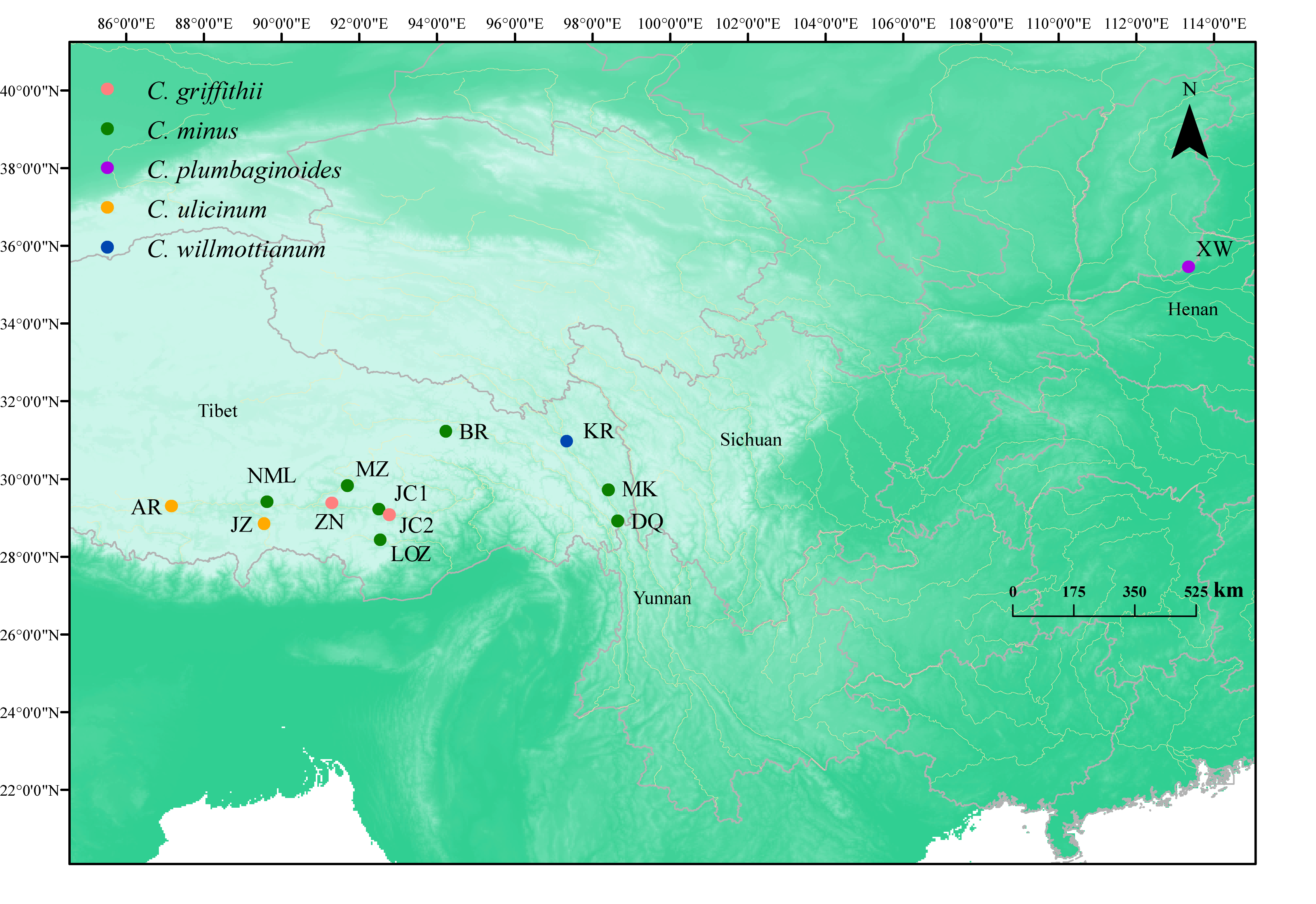

Supplement: Supplementary file 5 — Supplementary Material 5 [file 12870_2023_4323_MOESM5_ESM.tif]
