# Supplementary material for: Characteristics of plastid genomes in the genus Ceratostigma inhabiting arid habitats in China and their phylogenomic implications
Source: BMC Plant Biol. 2023 Jun 7;23:303. doi: 10.1186/s12870-023-04323-7 (PMC10245475; doi:10.1186/s12870-023-04323-7)
Supplement: Supplementary file 9 — Supplementary Material 9 [file 12870_2023_4323_MOESM9_ESM.docx]

Table S6 Sampling information with locality and voucher numbers of the five *Ceratostigma* species

| Locality ID | Species | Location | Voucher No. |
| --- | --- | --- | --- |
| ZN | *C*. *griffithii* | Zhanang, Tibet | ZYJ014 |
| JC2 | *C*. *griffithii* | Jiacha, Tibet | ZYJ006 |
| DQ | *C*. *minus* | Deqin, Yunnan | ZYJ044 |
| MK | *C*. *minus* | Mangkang, Tibet | ZYJ001 |
| NML | *C*. *minus* | Nanmulin, Tibet | GX026 |
| JC1 | *C*. *minus* | Jiacha, Tibet | ZYJ007 |
| LOZ | *C*. *minus* | Longzi, Tibet | ZYJ013 |
| BR | *C*. *minus* | Biru, Tibet | ZYJ034 |
| MZ | *C*. *minus* | Mozhugongka, Tibet | GX008 |
| XW1 | *C*. *plumbaginoides* | Xiuwu, Henan | XW001 |
| XW2 | *C*. *plumbaginoides* | Xiuwu, Henan | XW001 |
| AR | *C*. *ulicinum* | Angren, Tibet | GX025 |
| JZ | *C*. *ulicinum* | Jiangzi, Tibet | ZYJ015 |
| KR | *C*. *willmottianum* | Changdu, Tibet | GX047 |
