# Supplementary material for: Characteristics of plastid genomes in the genus Ceratostigma inhabiting arid habitats in China and their phylogenomic implications
Source: BMC Plant Biol. 2023 Jun 7;23:303. doi: 10.1186/s12870-023-04323-7 (PMC10245475; doi:10.1186/s12870-023-04323-7)
Supplement: Supplementary file 10 — Supplementary Material 10 [file 12870_2023_4323_MOESM10_ESM.docx]

Table S1 Protein-coding genes and functional groups in the sequences of *Ceratostigma* plastid genome

| Category of genes | Function of genes | Names of genes |
| --- | --- | --- |
| Subunits of ATP synthase (ATP) | Genes for photosynthesis | *atpA, atpB, atpE, atpF, atpH, atpI* |
| Subunits of NADH dehydrogenase (NDH) | Genes of photosynthesis | *ndhA, ndhB***, ndhC, ndhD, ndhE, ndhF,ndhG, ndhH,ndhI, ndhJ, ndhK* |
| Subunits of photosystem Ⅰ(PSA) | Genes of photosynthesis | *psaA, psaB, psaC, psaI, psaJ* |
| Subunits of photosystem Ⅱ(PSB) | Genes of photosynthesis | *psbA, psbB, psbC, psbD, psbE, psbF, psbH, psbI, psbJ, psbK, psbL, psbM, psbN, psbT* |
| Subunits of cytochrome (PET) |  | *petA, petB, petD, petG, petL, petN* |
| Large subunit of Rubisco (Rubisco) | Genes for photosynthesis | *rbcL* |
| Small subunit of ribosome (RPS) | Self-replication | *rps2, rps3, rps4, rps7***, rps8, rps11, rps12***, rps14, rps15, rps16, rps18, rps19* |
| DNA dependent RNA polymerase (RPO) | Self-replication | *rpoA, rpoB, rpoC1, rpoC2* |
| Large subunit of ribosome (RPL) | Self-replication | *rpl2#, rpl14, rpl16, rpl20, rpl22, rpl32, rpl33, rpl36* |
| other genes (OG) |  |  |
| Conserved open reading frames | Genes of unknown function | *ycf1***, ycf2***, ycf3, ycf4* |
| Subunits of acetyl-CoA |  | *accD* |
| C-type cytochrome synthesis gene |  | *ccsA* |
| Envelope membrane protein |  | *cemA* |
| Translational initiation factor |  | *infA* |
| Maturase |  | *matK* |

Genes with * are those duplicated genes in invented repeat regions and the gene *rpl2* # has two copies in *C. ulicinum* in invented repeat regions but one copy in *C. minus, C. griffithii, C. willmottianum* and *C. plumbaginoides* in the large single copy regions.
