# Supplementary material for: Characteristics of plastid genomes in the genus Ceratostigma inhabiting arid habitats in China and their phylogenomic implications
Source: BMC Plant Biol. 2023 Jun 7;23:303. doi: 10.1186/s12870-023-04323-7 (PMC10245475; doi:10.1186/s12870-023-04323-7)
Supplement: Supplementary file 12 — Supplementary Material 12 [file 12870_2023_4323_MOESM12_ESM.docx]

Table S7 List of accession with GenBank accession number, length and references included in the phylogenetic analysis.

| Family/subfamily/tribe | Species | GenBank No. | length of the genome sequenced (bp) | references |
| --- | --- | --- | --- | --- |
| Plumbaginaceae/ Plumbagineae | *Cerotastigma* *willmottianum* | MK397862 | 164,999 | Yao et al., 2019 |
| Plumbaginaceae/ Plumbagineae | *Plumbago auriculata* | MK397887 | 169,389 | Yao et al., 2019 |
| Plumbaginaceae / Staticeae | *Limonium tetragonum* | MN044572 | 154,789 | Kim et al., 2021 |
| Plumbaginaceae/ Staticeae | *Limonium bicolor* | NC_059915 | 154,617 | Darshetkar et al., 2021 |
| Plumbaginaceae/ Staticeae | *Limonium tenellum* | MK397871 | 150,515 | Yao et al., 2019 |
| Plumbaginaceae/ Staticeae | *Limonium sinensis* | MN599096 | 174,033 | Li et al., 2020 |
| Plumbaginaceae/ Staticeae | *Limonium aureum* | MN623109 | 154,661 | Zhang et al., 2020 |
| Polygonaceae/Polygonoideae | *Oxyria sinensis* | MK397882 | 156,034 | Yao et al., 2019 |
| Polygonaceae/Polygonoideae | *Rheum palmatum* | NC_027728 | 161,541 | Yao et al., 2019 |
| Polygonaceae/Polygonoideae  Cactaceae/Opuntioideae  Cactaceae/Opuntioideae  Cactaceae/Opuntioideae | *Fallopia multiflora*  *Opuntia auberi*  *Opuntia gaumeri*  *Opuntia retrorsa* | MK330002  MZ366771  MZ366779  MZ366785 | 163,773  6433  6433  6434 | Yao et al., 2019  Kohler et al., 2021  Kohler et al., 2021  Kohler et al., 2021 |

Note: The three sequences of *Opuntia* were used for phylogenetic analyses based on the nuclear ribosome DNA data and others for phylogenetic analyses based on plastid genomes.

**References**

Darshetkar AM, Maurya S, Lee C et al. (2021) Plastome analysis unveils Inverted Repeat (IR) expansion and positive selection in Sea Lavenders (*Limonium*, Plumbaginaceae, Limonioideae, Limonieae). ***Phytokeys***:89-107

Li JF, Xu B, Yang Q et al. (2020) The complete chloroplast genome sequence of *Limonium sinense* (Plumbaginaceae). ***Mitochondrial DNA Part B-Resources*** 5:556-557

Kim Y, Xi H, Park J (2021) The complete chloroplast genome of *Limonium tetragonum* (Plumbaginaceae) isolated in Korea. ***Korean Journal of plant Taxonomy*** 51:337-344

Kohler M, Oakley LJ, Font F et al. (2021) On the continuum of evolution: a putative new hybrid speciation event in *Opuntia* (Cactaceae) between a native and an introduced species in southern South America. ***Systematics and Biodiversity*** 19:1026-1039

Yao G, Jin JJ, Li HT et al. (2019) Plastid phylogenomic insights into the evolution of Caryophyllales. ***Molecular Phylogenetics and Evolution*** 134:74-86

Zhang XY, Xu Y, Liu X (2020) Complete plastome sequence of *Limonium aureum*, a medicinal and ornamental species in China. ***Mitochondrial DNA Part B-Resources*** 5:333-334
