# Supplementary material for: Characteristics of plastid genomes in the genus Ceratostigma inhabiting arid habitats in China and their phylogenomic implications
Source: BMC Plant Biol. 2023 Jun 7;23:303. doi: 10.1186/s12870-023-04323-7 (PMC10245475; doi:10.1186/s12870-023-04323-7)
Supplement: Supplementary file 13 — Supplementary Material 13 [file 12870_2023_4323_MOESM13_ESM.docx]

**Supplementary Information**

**Additional file 1: Fig. S1.** Plastid genome maps of the five *Ceratostigma* species. The genes drawn outside the circle are transcribes clockwise, and those outside are counterclockwise. Large single copy (LSC), small single copy (SSC) and inverted repeats (IRA and IRB) are indicated. The innermost darker gray represents the GC content of the plastomes. Genes belonging to different functional groups are color-coded.

**Additional file 2: Fig. S2.** The phylogenetic trees of all accessions based on plastid genome sequences after allowing all (A) and none (B) gap positions using maximum likelihood (ML) and Bayesian inference (BI) analyses. The number above lines indicates bootstrap values for ML and posterior probabilities for BI of the phylogenetic analysis for each clade.

**Additional file 3: Fig. S3.** The phylogenetic trees of all accessions based on the nuclear ribosomal DNA (nrDNA) data using maximum likelihood (ML) (A) and Bayesian inference (BI) (B) analyses. The number above lines indicates bootstrap values for ML and posterior probabilities for BI of the phylogenetic analysis for each clade.

**Additional file 4: Fig. S4.** The phylogenetic trees of all accessions based on the combined plastid genome data and the nuclear ribosomal DNA (nrDNA) data using maximum likelihood (ML) (A) and Bayesian inference (BI) (B) analyses. The number above lines indicates bootstrap values for ML and posterior probabilities for BI of the phylogenetic analysis for each clade.

**Additional file 5: Fig. S5.** Geographical distribution and sampling locations of *Ceratostigma* species.

**Additional file 6: Table S1.** Plastid genes and functional groups in the sequences *Ceratostigma* chloroplast genome.

**Additional file 7: Table S2.** The nucleotide diversity (Pi) of the coding regions and the non-coding regions of five *Cerotastigma* plastomes.

**Additional file 8: Table S3.** Simple sequence repeats (SSRs) distribution in the the five *Cerotastigma* plastomes.

**Additional file 9: Table S4.** Codon usage and relative synonymous codon usage (RSCU) values of protein-coding genes of the five *Cerotastigma*.

**Additional file 10: Table S5.** Results of selective pressure analyses in PALM with the site-specific model.

**Additional file 11: Table S6.** Sampling information with locality and voucher numbers of the five *Ceratostigma* species

**Additional file 12: Table S7.** List of accession with GenBank accession number, length and references included in the phylogenetic analysis.
